# Facile Synthesis and Antimicrobial Evaluation of Some New Heterocyclic Compounds Incorporating a Biologically Active Sulfamoyl Moiety

**DOI:** 10.1155/2014/165495

**Published:** 2014-08-17

**Authors:** Elham S. Darwish

**Affiliations:** Department of Chemistry, Faculty of Science, Cairo University, Giza 12613, Egypt

## Abstract

A facile and convenient synthesis of new heterocyclic compounds containing a sulfamoyl moiety suitable for use as antimicrobial agents was reported. The precursor 3-oxo-3-phenyl-*N*-(4-sulfamoylphenyl)propionamide was coupled smoothly with arenediazonium salt producing hydrazones which reacted with malononitrile or triethylorthoformate affording pyridazine and triazine derivatives, respectively. Also, the reactivity of the same precursor with DMF-DMA was followed by aminotriazole; aromatic aldehydes was followed by hydrazine hydrate, triethylorthoformate, or thiourea affording triazolo[1,5-a]pyrimidine, pyrazole, acrylamide, and dihydropyrimidine derivatives, respectively. On the other hand, treatment of the precursor propionamide with phenyl isothiocyanate and KOH in DMF afforded the intermediate salt which was treated with dilute HCl followed by 2-bromo-1-phenylethanone affording carboxamide derivative. While the same intermediate salt reacted *in situ* with chloroacetone, ethyl 2-chloroacetate, 3-(2-bromoacetyl)-2*H*-chromen-2-one, methyl iodide, or 2-oxo-*N*-phenylpropane hydrazonoyl chloride afforded the thiophene, ketene *N*,*S*-acetal, and thiadiazole derivatives, respectively. The structure of the new products was established based on elemental and spectral analysis. Antimicrobial evaluation of some selected examples from the synthesized products was carried out whereby four compounds were found to have moderate activities and one compound showed the highest activity.

## 1. Introduction

Sulfonamides have been reported to exhibit antimicrobial [1–3], antifungal [4], insulin releasing [5], carbonic anhydrase inhibitory [6], anti-inflammatory [7], and antitumor [8], properties. Some active sulfonamides as antibacterial are also known for their immune modifying effects [9]. Also, pyrazole derivatives are known to exhibit diverse bioactivities such as antidepressant [10], anticonvulsant [11], antimicrobial [12], analgesic [13], and antitumor [14] activity and also serve as human acyl-CoA: cholesterol acyltransferase inhibitors [15]. In addition, thiophene compounds are well known to exhibit various biological and medicinal activities such as BACEI inhibitors [16], antitubercular [17], antidepressant [18], anti-inflammatory [19], and anti-HIV PR inhibitors [20], and antibreast cancer activities [21]. These facts, coupled with our desire to develop efficacious antimicrobial agents, and in continuation of our work in heterocycles of biological interest [22–25], prompted us to devise an efficient and convenient method of synthesis of hitherto unknown and novel hydrazone, pyridazine, acrylamide, pyrazole, triazolo[1,5-a]pyrimidine, thiadiazole, dihydropyrimidine, and thiophene derivatives with a sulfonamide nucleus. Results from assessment of the antimicrobial activity of these newly synthesized compounds are reported in this study.

## 2. Experimental Part

All melting points were determined on an electrothermal Gallenkamp apparatus and are uncorrected. The IR spectra were measured on a Pye-Unicam SP300 instrument in potassium bromide discs. The ^1^H-NMR spectra were recorded on a Varian Mercury VXR-300 spectrometer (300 MHz). The mass spectra were recorded on a GCMS-Q1000-EX Shimadzu and GCMS 5988-A HP spectrometers; the ionizing voltage was 70 eV. Elemental analyses were carried out by the Microanalytical Center of Cairo University, Giza, Egypt. The biological evaluation of the products** 3a**,** 3c**,** 6**,** 7**,** 8b**,** 10**,** 15**,** 16,** and** 17** was carried out at the Medical Mycology Laboratory of the Regional Center for Mycology and Biotechnology of Al-Azhar University, Cairo, Egypt.

3-Oxo-3-phenyl-*N*-(4-sulfamoylphenyl)propionamide (**1**) and 3-(2-bromoacetyl)-2*H*-chromen-2-one [26–29] were prepared as previously described.

### 2.1. Coupling of 3-Oxo-3-phenyl-N-(4-sulfamoylphenyl)propionamide (**1**) with the Appropriate Diazonium Salt of Aromatic Amines

*General Procedure.* To a cold solution of 3-oxo-3-phenyl-*N*-(4-sulfamoylphenyl)propionamide (**1**) (0.318 g, 1 mmol) in pyridine (20 mL), the appropriate diazonium salt of aromatic amine (aniline or 4-methylaniline or 4-chloroaniline or 4-methoxy-aniline or 4-nitroaniline) (1 mmol) was added (prepared according to literature procedures) [30]. The addition was carried out portionwise with stirring at 0–5°C over a period of 30 min. After complete addition, the reaction mixture was stirred for further 4 hrs, then kept in an ice chest for 12 hrs, and finally diluted with water. The precipitated solid was collected by filtration, washed with water, dried, and finally recrystallized from the proper solvent to afford the corresponding coupling products** 3a**–**e**.

### 2.2. 2-[(4-Methoxyphenyl)hydrazono]-3-oxo-3-phenyl-N-(4-sulfamoylphenyl)propionamide (**3a**)

Yield (80%), m.p. 270°C (dioxane); IR (KBr) *ν*_max⁡_: 3351, 3264 (NH, NH_2_), 3073 (aromatic CH), 1657 (C=O) cm^−1^; ^1^H NMR (DMSO-d_6_): *δ* 3.75 (s, 3H, OCH_3_), 6.96 (d, 2H, *J* = 9 Hz), 7.28 (d, 2H, *J* = 9 Hz), 7.30 (s, 2H, D_2_O-exchangeable NH_2_), 7.61 (d, 2H, *J* = 8 Hz), 7.77 (d, 2H, *J* = 8 Hz), 7.79–7.93 (m, 5H, Ar-H), 11.47 (s, 1H, D_2_O-exchangeable NH), 13.82 (s, 1H, D_2_O-exchangeable NH); MS* m/z* (%): 452 (M^+^+2, 3.33), 452 (M^+^, 18.37), 347 (4.07), 252 (15.66), 196 (3.51), 172 (5.49), 105 (100.0). Anal. Calcd for C_22_H_20_N_4_O_5_S (452.49): C, 58.40; H, 4.46; N, 12.38; S, 7.09. Found: C, 58.36; H, 4.36; N, 12.32; S, 7.01%.

### 2.3. 2-(p-Tolylhydrazono)-3-oxo-3-phenyl-N-(4-sulfamoylphenyl)propionamide (**3b**)

Yield (75%), m.p. 280°C (dioxane); IR (KBr) *ν*_max⁡_: 3352, 3265 (NH, NH_2_), 2924, 2858 (aliphatic CH), 1660 (C=O) cm^−1^; ^1^H NMR (DMSO-d_6_): *δ* 2.26 (s, 3H, CH_3_), 7.14 (s, 2H, D_2_O-exchangeable NH_2_), 7.17–7.30 (m, 5H, Ar-H), 7.56 (d, 2H, *J* = 9 Hz), 7.62 (d, 2H, *J* = 9 Hz), 7.81 (d, 2H, *J* = 8 Hz), 7.93 (d, 2H, *J* = 8 Hz), 11.37 (s, 1H, D_2_O-exchangeable NH), 13.55 (s, 1H, D_2_O-exchangeable NH); MS* m/z* (%): 437 (M^+^+1, 25.12), 436 (M^+^, 35.75), 332 (26.57), 329 (32.85), 279 (51.21), 263 (33.82), 171 (42.03), 157 (31.40), 104 (28.02), 57 (100.0). Anal. Calcd for C_22_H_20_N_4_O_4_S (436.49): C, 60.54; H, 4.62; N, 12.84; S, 7.35. Found: C, 60.50; H, 4.55; N, 12.72; S, 7.27%.

### 2.4. 2-(Phenylhydrazono)-3-oxo-3-phenyl-N-(4-sulfamoylphenyl)propionamide (**3c**)

Yield (70%), m.p. 282°C (dioxane); IR (KBr) *ν*_max⁡_: 3349, 3256 (NH, NH_2_), 3059 (aromatic CH), 1650 (C=O) cm^−1^; ^1^H NMR (DMSO-d_6_): *δ* 7.18 (s, 2H, D_2_O-exchangeable NH_2_), 7.20–7.66 (m, 5H, Ar-H), 7.70 (d, 2H, *J* = 9 Hz), 7.76 (d, 2H, *J* = 9 Hz), 7.85–8.00 (m, 5H, Ar-H), 11.59 (s, 1H, D_2_O-exchangeable NH), 13.46 (s, 1H, D_2_O-exchangeable NH); MS* m/z* (%): 422 (M^+^, 7.6), 223 (1.9), 199 (0.4), 171 (1.8), 121 (20.3), 119 (3.0), 105 (100.0), 77 (57.7). Anal. Calcd for C_21_H_18_N_4_O_4_S (422.47): C, 59.71; H, 4.29; N, 13.26; S, 7.59. Found: C, 59.66; H, 4.26; N, 13.30; S, 7.53%.

### 2.5. 2-[(4-Chlorophenyl)hydrazono]-3-oxo-3-phenyl-N-(4-sulfamoylphenyl)propionamide (**3d**)

Yield (60%), m.p. 304°C (dioxane); IR (KBr) *ν*_max⁡_: 3364, 3262 and 3152 (NH, NH_2_), 1660 (C=O) cm^−1^; ^1^H NMR (DMSO-d_6_): *δ* 7.24 (s, 2H, D_2_O-exchangeable NH_2_), 7.52 (d, 2H, *J* = 8 Hz), 7.62 (d, 2H, *J* = 8 Hz), 7.64 (d, 2H, *J* = 9 Hz), 7.83 (d, 2H, *J* = 9 Hz), 7.85–7.93 (m, 5H, Ar-H), 11.21 (s, 1H, D_2_O-exchangeable NH), 13.05 (s, 1H, D_2_O-exchangeable NH); MS* m/z* (%): 457 (M^+^+1, 13.25), 456 (M^+^, 16.64), 329 (8.10), 285 (11.19), 199 (10.90), 128 (11.49), 105 (100.0). Anal. Calcd for C_21_H_17_ClN_4_O_4_S (456.91): C, 55.20; H, 3.75; Cl, 7.76; N, 12.26; S, 7.02. Found: C, 55.15; H, 3.66; Cl, 7.66; N, 12.21; S, 7.00%.

### 2.6. 2-[(4-Nitrophenyl)hydrazono]-3-oxo-3-phenyl-N-(4-sulfamoylphenyl)propionamide (**3e**)

Yield (55%), m.p. 302°C (dioxane); IR (KBr) *ν*_max⁡_: 3368, 3265 and 3161 (NH, NH_2_), 1664 (C=O) cm^−1^; ^1^H NMR (DMSO-d_6_): *δ* 7.26 (s, 2H, D_2_O-exchangeable NH_2_), 7.59 (d, 2H, *J* = 9 Hz), 7.70 (d, 2H, *J* = 9 Hz), 7.78–7.89 (m, 5H, Ar-H), 7.91 (d, 2H, *J* = 8 Hz), 8.20 (d, 2H, *J* = 8 Hz), 11.08 (s, 1H, D_2_O-exchangeable NH), 12.45 (s, 1H, D_2_O-exchangeable NH); MS* m/z* (%):468 (M^+^+1, 3.89), 467 (M^+^, 7.25), 362 (4.71), 296 (4.26), 199 (4.33), 172 (19.88), 105 (100.0). Anal. Calcd for C_21_H_17_N_5_O_6_S (467.46): C, 53.96; H, 3.67; N, 14.98; S, 6.86. Found: C, 53.87; H, 3.59; N, 14.88; S, 6.78%.

### 2.7. N-[4-(Aminosulfonyl)phenyl]-5-cyano-6-imino-4-phenyl-p-tolyl-1,6-dihydropyridazine-3-carboxamide (**4**)

To a solution of (**3b**) (0.436 g, 1 mmol) and malononitrile (1 mmol) in dioxane (20 mL), few drops of piperidine were added and the reaction mixture was refluxed for 6 hrs. The solid product that formed was filtered off, washed with ethanol, and then recrystallized from the proper solvent to give** 4**.

Yield (55%), m.p. > 300°C (DMF); IR (KBr) *ν*_max⁡_: 3462, 3305 and 3181 (NH, NH_2_), 2203 (C*≡*N), 1637 (C=O) cm^−1^; ^1^H NMR (DMSO-d_6_): *δ* 2.49 (s, 3H, CH_3_), 7.32 (d, 2H D_2_O-exchangeable NH_2_), 7.40 (d, 2H, *J* = 8 Hz), 7.59 (d, 2H, *J* = 9 Hz), 7.67–7.90 (m, 9H, Ar-H), 11.20 (s, 1H, D_2_O-exchangeable NH), 14.60 (s, 1H, D_2_O-exchangeable NH); MS* m/z* (%): 484 (M^+^, 2.32), 409 (7.42), 393 (9.09), 313 (6.94), 285 (6.58), 199 (7.89), 91 (16.51), 77 (26.56), 69 (100.0). Anal. Calcd for C_25_H_20_N_6_O_3_S (484.54): C, 61.97; H, 4.16; N, 17.34; S, 6.62. Found: C, 61.92; H, 4.11; N, 17.30; S, 6.59%.

### 2.8. 4-(6-Benzoyl-3-ethoxy-5-oxo-2-p-tolyl-2,3-dihydro-1,2,4-triazin-4-(5H)-yl)benzenesulfonamide (**5**)

To a solution of the compound** 3b** (0.436 g, 1 mmol) in acetic acid (20 mL), triethyl orthoformate (0.2 mL, 1 mmol) was added and the reaction mixture was refluxed for 8 hrs; then it was left to cool. So the solid product formed was filtered off, washed with EtOH, and dried. Recrystallization from dioxane afforded 4-(6-benzoyl-3-ethoxy-5-oxo-2-*p*-tolyl-2,3-dihydro-1,2,4-triazin-4-(5*H*)-yl)benzenesulfonamide (**5**).

Yield (50%), m.p. 296°C (DMF); IR (KBr) *ν*_max⁡_: 3370, 3311 and 3236 (NH, NH_2_), 3056 (aromatic CH), 2982 (aliphatic-H), 1678 (C=O) cm^−1^; ^1^H NMR (DMSO-d_6_): *δ* 1.32 (t, 3H, *J* = 7.2 Hz, CH_3_), 2.24 (s, 3H, CH_3_), 4.27 (q, 2H, *J* = 7.2, CH_2_), 5.94 (S, 1H), 7.36 (s, 2H, D_2_O-exchangeable NH_2_), 7.37–7.57 (m, 9H, Ar-H), 7.60 (d, 2H, *J* = 9 Hz), 7.71 (d, 2H, *J* = 9 Hz); MS* m/z* (%): 494 (M^+^+2, 0.1), 492 (M^+^, 0.5), 352 (0.42), 335 (1.6), 156 (0.6), 105 (100.0), 91 (1.5). Anal. Calcd for C_25_H_24_N_4_O_5_S (492.56): C, 60.96; H, 4.91; N, 11.37; S, 6.51. Found: C, 60.88; H, 4.85; N, 11.31; S, 6.50%.

### 2.9. 2-Benzoyl-3-dimethylamino-N-(4-sulfamoylphenyl)acrylamide (**6**)

A mixture of the compound** 1** (3.18 g, 10 mmol) and* N*,*N*-dimethylformamide dimethyl acetal (DMF-DMA) (10 mmol) in dry dioxane (30 mL) was refluxed for 6 hrs; then it was left to cool at room temperature. The yellow precipitated product was filtered off, washed with petroleum ether, and dried. Crystallization from MeOH was carried out to give 2-benzoyl-3-dimethylamino-*N*-(4-sulfamoylphenyl)acryl-amide (**6**) in 44% yield.

Yield (44%), m.p. 190°C (MeOH); IR (KBr) *ν*_max⁡_: 3250, 3112 (NH, NH_2_), 3056 (aromatic CH), 1683 (C=O), cm^−1^; ^1^H NMR (DMSO-d_6_): *δ* 3.42 (s, 6H, 2CH_3_), 6.62 (d, 2H, *J* = 9 Hz), 7.03 (s, 2H, D_2_O-exchangeable NH_2_), 7.28–7.31 (m, 5H, Ar-H), 7.58 (d, 2H, *J* = 9 Hz), 8.10 (s, 1H, olefinic-H), 9.98 (s, 1H, D_2_O-exchangeable NH); MS* m/z* (%): 373 (M^+^, 0.4), 291 (1.1), 190 (0.5), 155 (16.3), 105 (100.0). Anal. Calcd for C_18_H_19_N_3_O_4_S (373.43): C, 57.90; H, 5.13; N, 11.25; S, 8.59. Found: C, 57.89; H, 5.03; N, 11.15; S, 8.61%.

### 2.10. 5-Phenyl-[1,2,4]triazolo[1,5-a]pyrimidine-6-carboxylic Acid (4-sulfamoylphenyl)amide (**7**)

To a solution of the compound** 6** (0.37 g, 1 mmol) in acetic acid (20 mL), amino triazolo (0.1 g, 1 mmol) was added and the reaction mixture was refluxed for 8 hrs; then it was left to cool. The solid product formed was filtered off, washed with EtOH, and dried. Recrystallization from dioxane afforded 5-phenyl-[1,2,4]triazolo[1,5-a]-pyrimidine-6-carboxylic acid(4-sulfamoylphenyl)amide (**7**).

Yield (48%), m.p. 290°C (dioxane); IR (KBr) *ν*_max⁡_: 3261, 3110 (NH, NH_2_), 1685 (C=O), cm^−1^; ^1^H NMR (DMSO-d_6_): *δ* 6.61 (s, 1H, D_2_O-exchangeable NH_2_), 7.05 (d, 2H, *J* = 9 Hz), 7.34–7.44 (m, 5H, Ar-H), 7.50 (d, 2H, *J* = 9 Hz), 7.68 (s, 1H, triazole-H), 8.09 (s, 1H, pyrimidine-H), 8.48 (s, 1H, D_2_O-exchangeable NH); MS* m/z* (%): 397 (M^+^+3, 20.0), 394 (M+, 0.2), 157 (16.0), 121 (52.0), 76 (84.0), 63 (100.0). Anal. Calcd for C_18_H_14_N_6_O_3_S (394.41): C, 54.82; H, 3.58; N, 21.31; S, 8.13. Found: C, 54.80; H, 3.53; N, 21.25; S, 8.01%.

### 2.11. 2-Benzoyl-3-aryl-2-yl-N-(4-sulfamoylphenyl)acrylamide (**8a, b**)

*General Procedure.* To a solution of (**1**) (0.318 g, 1 mmol) and the appropriate aromatic aldehydes (1 mmol) in dioxane (20 mL), few drops of piperidine were added and the reaction mixture was refluxed for 6 hrs. So the solid product formed was filtered off, washed with EtOH, dried, and finally recrystallized from the proper solvent to give** 8a**,** b**.

### 2.12. 2-Benzoyl-3-furan-2-yl-N-(4-sulfamoylphenyl)acrylamide (**8a**)

Yield (45%), m.p. 220°C (EtOH); IR (KBr) *ν*_max⁡_: 3349, 3255 (NH, NH_2_), 1687 (C=O) cm^−1^; ^1^H NMR (DMSO-d_6_): *δ* 7.43 (s, 2H, D_2_O-exchangeable NH_2_), 7.46–7.61 (m, 8H, Ar-H), 7.64 (d, 2H, *J* = 9 Hz), 7.94 (d, 2H, *J* = 9 Hz), 8.48 (s, 1H, olefinic-H), 9.14 (s, 1H, D_2_O-exchangeable NH); MS* m/z* (%): 396 (M^+^, 0.1), 225 (0.3), 197 (0.4), 171 (22.4), 157 (2.8), 105 (100.0). Anal. Calcd for C_20_H_16_N_2_O_5_S (396.42): C, 60.60; H, 4.07; N, 7.07; S, 8.09. Found: C, 60.53; H, 4.01; N, 7.11; S, 8.00%.

### 2.13. 2-Benzoyl-3-(1,3-diphenyl-1H-pyrazol-4-yl)-N-(4-sulfamoylphenyl)acrylamide (**8b**)

Yield (50%), m.p. 180°C (EtOH); IR (KBr) *ν*_max⁡_: 3341, 3261 (NH, NH_2_), 3060 (aromatic CH), 1675 (C=O) cm^−1^; ^1^H NMR (DMSO-d_6_): *δ* 7.25 (s, 2H, D_2_O-exchangeable NH_2_), 7.49–7.00 (m, 15H, Ar-H), 7.64 (s, 1H, pyrazole-H), 7.77 (d, 2H, *J* = 9 Hz), 7.81 (d, 2H, *J* = 9 Hz), 7.86 (s, 1H, olefinic-H), 10.85 (s, 1H, D_2_O-exchangeable NH); MS* m/z* (%): 548 (M^+^, 0.1), 217 (16.7), 199 (20.0), 172 (16.7), 105 (96.7), 92 (20.0), 77 (100.0). Anal. Calcd for C_31_H_24_N_4_O_4_S (548.63): C, 67.87; H, 4.41; N, 10.21; S, 5.84. Found: C, 67.82; H, 4.35; N, 10.16; S, 5.78%.

### 2.14. 5-Furan-2-yl-3-phenyl-2,3-dihydro-1-H-pyrazole-4-carboxylic Acid (4-sulfamoylphenyl)amide (**9a**)

To a solution of the compound** 8a** (0.4 g, 1 mmol) in dioxane (20 mL), hydrazine hydrate (80%, 0.2 mL, 1 mmol) was added and the reaction mixture was refluxed for 6 hrs; then it was left to cool. So the solid product formed was filtered off, washed with EtOH, and dried. Recrystallization from dioxane afforded** 9a**.

Yield (40%), m.p. 244°C (EtOH); IR (KBr) *ν*_max⁡_: 3281, 3111 (NH, NH_2_), 1666 (C=O) cm^−1^; ^1^H NMR (DMSO-d_6_): *δ* 6.33–6.37 (m, 2H), 7.21 (s, 2H, D_2_O-exchangeable NH_2_), 7.44 (d, 2H, *J* = 9 Hz), 7.50 (d, 2H, *J* = 9 Hz), 7.56–7.75 (m, 6H, Ar-H), 9.99 (s, 1H, D_2_O-exchangeable NH), 10.42 (s, 1H, D_2_O-exchangeable NH); MS* m/z* (%): 408 (M^+^, 3.5), 171 (20.4), 142 (3.5), 77 (100). Anal. Calcd for C_20_H_16_N_4_O_4_S (408.44): C, 58.81; H, 3.95; N, 13.72; S, 7.85. Found: C, 58.79; H, 3.93; N, 13.65; S, 7.80%.

### 2.15. 2-Benzoyl-3-ethoxy-N-(4-sulfamoylphenyl)acrylamide (**10**)

To a solution of the compound** 1** (3.18 g, 10 mmol) in acetic anhydride (20 mL), triethyl orthoformate (2 mL, 10 mmol) was added and the reaction mixture was refluxed for 8 hrs; then it was left to cool. So the solid product formed was filtered off, washed with EtOH, and dried. Recrystallization from dioxane afforded 2-benzoyl-3-ethoxy-*N*-(4-sulfamoylphenyl)acrylamide (**10**).

Yield (60%), m.p. 280°C (DMF); IR (KBr) *ν*_max⁡_: 3359, 3239 and 3107 (NH, NH_2_), 1655 (C=O) cm^−1^; ^1^H NMR (DMSO-d_6_): *δ* 1.26 (t, 3H, *J* = 7.2 Hz, CH_3_), 4.32 (q, 2H, *J* = 7.2, CH_2_), 7.16 (s, 2H, D_2_O-exchangeable NH_2_), 7.40 (d, 2H, *J* = 9 Hz), 7.69 (d, 2H, *J* = 9 Hz), 7.87–8.00 (m, 5H, Ar-H), 8.63 (s, 1H, CH), 11.25 (s, 1H, D_2_O-exchangeable NH); MS* m/z* (%): 374 (M^+^, 0.5), 370 (0.6), 301 (72.7), 198 (18.0), 172 (33.6), 156 (59.6), 105 (70.8), 77 (85.0), 65 (100.0). Anal. Calcd for C_18_H_18_N_2_O_5_S (374.42): C, 57.74; H, 4.85; N, 7.48; S, 8.56. Found: C, 57.70; H, 4.76; N, 7.41; S, 8.50%.

### 2.16. 4-(6-Phenyl-2-thioxo-2,3-dihydropyrimidin-4-ylamino)benzenesulfonamide (**11**)

To a mixture of** 1** (0.318 g, 1 mmol) and thiourea (0.076 g, 1 mmol) in dimethylformamide (20 mL), triethylamine (0.5 mL) was added and the reaction mixture was refluxed for 6 hrs; then it was left to cool. The precipitated product was filtered off and purified by recrystallization from the suitable solvent to afford the corresponding** 11**.

Yield (45%), m.p. > 300°C (dioxane); IR (KBr) *ν*_max⁡_: 3500–3368 (NH, NH_2_), 3057 (aromatic CH), 1675 (C=O) cm^−1^; ^1^H NMR (DMSO-d_6_): *δ* 7.27 (s, 2H, D_2_O-exchangeable NH_2_), 7.54 (d, 2H, *J* = 9 Hz), 7.66 (d, 2H, *J* = 9 Hz), 7.68–7.97 (m, 6H, Ar-H), 11.85 (s, 1H, D_2_O-exchangeable NH), 12.23 (s, 1H, D_2_O-exchangeable NH). Anal. Calcd for C_16_H_14_N_4_O_2_S_2_ (358.44): C, 53.61; H, 3.94; N, 15.63; S, 17.89. Found: C, 53. 60; H, 3.90; N, 15.59; S, 17.84.%.

### 2.17. 2-Benzoyl-3-mercapto-3-phenylamino-N-(4-sulfamoylphenyl)acrylamide (**13**)

To a stirred solution of potassium hydroxide (0.11 g, 1 mmol) in dimethylformamide (20 mL) the** 1** (0.318 g, 1 mmol) was added. After stirring for 30 min, phenylisothiocyanate (0.27 g, 0.24 mL, 1 mmol) was added to the resulting mixture and stirring was continued for 6 h; then it was poured over crushed ice containing hydrochloric acid. The solid product formed was filtered off, washed with water, dried, and finally recrystallized from dioxane to afford** 13**.

Yield (40%), m.p. 230°C (dioxane); IR (KBr) *ν*_max⁡_: 3350, 3256 (NH, NH_2_), 1689 (C=O), 1661 (C=O), cm^−1^; ^1^H NMR (DMSO-d_6_): *δ* 7.19 (s, 2H, D_2_O-exchangeable NH_2_), 7.39 (d, 2H, *J* = 9 Hz), 7.69 (d, 2H, *J* = 9 Hz), 7.71–7.90 (m, 10H, Ar-H), 10.39 (s, 1H, D_2_O-exchangeable NH), 11.13 (s, 1H, D_2_O-exchang-eable NH), 12.76 (s, 1H, SH); MS* m/z* (%): 455 (M^+^+2, 26.3), 453 (M^+^, 1.5), 121 (26.3), 105 (100.0), 91 (47.4), 89 (36.8), 77 (89.5). Anal. Calcd for C_22_H_19_N_3_O_4_S_2_ (453.54): C, 58.26; H, 4.22; N, 9.26; S, 14.14. Found: C, 58.22; H, 4.20; N, 9.20; S, 14.11%.

### 2.18. N-[4-(Aminosulfonyl)phenyl]-2-anilino-4-phenyl-thiophene-5-benzoyl-3-carboxamide (**14**)

To a mixture of acrylamide** 13** (1 mmol) and 2-bromo-1-phenylethanone (0.2 g, 1 mmol) in dimethylformamide (20 mL), triethylamine (0.5 mL) was added and the reaction mixture was refluxed for 6 hrs; then it was left to cool. The precipitated product was filtered off and purified by recrystallization from dioxane to afford the corresponding* N*-[4-(aminosulfonyl)phenyl]-2-anilino-4-phenyl-thiophene-5-benzoyl-3-carboxamide (**14**).

Yield (40%), m.p. 280°C (dioxane); IR (KBr) *ν*_max⁡_: 3425, 3276, 3111 (NH, NH_2_), 3059 (aromatic CH), 1698 (C=O), 1658 (C=O), cm^−1^; ^1^H NMR (DMSO-d_6_): *δ* 7.19 (s, 2H, D_2_O-exchangeable NH_2_), 7.27–7.73 (m, 15H, Ar-H), 7.76 (d, 2H, *J* = 9 Hz), 8.22 (d, 2H, *J* = 9 Hz), 9.55 (s, 1H, D_2_O-exchangeable NH), 9.98 (s, 1H, D_2_O-exchangeable NH); MS* m/z* (%): 555 (M^+^+2, 16.0), 553 (M^+^, 1.5), 450 (20.0), 395 (20.0), 198 (24), 171 (36.0), 156 (24.0), 105 (92.0), 91 (16.0), 76 (20.0). Anal. Calcd for C_30_H_23_N_3_O_4_S_2_ (553.66): C, 65.08; H, 4.19; N, 7.59; S, 11.58. Found: C, 65.02; H, 4.15; N, 7.55; S, 11.54%.

### 2.19. Synthesis of **15**, **16**, **17**, **18**, and **20**

To a stirred solution of potassium hydroxide (0.11 g, 1 mmol) in DMF (20 mL), compound** 1** (0.318 g, 1 mmol) was added. After stirring for 30 min, phenyl isothiocyanate (0.27 g, 1 mmol) was added to the resulting mixture. Stirring was continued for 6 hrs, and then chloroacetone, ethyl chloroacetate, 3-(2-bromoacetyl)-2*H*-chromen-2-one, methyl iodide, or 2-oxo-*N*-phenylpropane hydrazonoyl chloride (1 mmol) was added portionwise over a period of 30 min. After the addition was complete, the reaction mixture was stirred for additional 12 h, during which the reactant dissolved and a yellow product precipitated. The solid product was filtered off, washed with EtOH, and dried; recrystallization from proper solvent afforded** 15**,** 16**,** 17**,** 18,** and** 20**.

### 2.20. N-[4-(Aminosulfonyl)phenyl]-2-anilino-4-phenyl-thiophene-5-acetyl-3-carboxamide (**15**)

Yield (65%), m.p. 268°C (dioxane); IR (KBr) *ν*_max⁡_: 3371, 3289 and 3220 (NH, NH_2_), 3059 (aromatic CH), 1636 (C=O), cm^−1^; ^1^H NMR (DMSO-d_6_): *δ* 1.72 (s, 3H, CH_3_), 7.11 (s, 2H, D_2_O-exchangeable NH_2_), 7.13 (d, 2H, *J* = 9 Hz), 7.38–7.46 (m, 10H, Ar-H), 7.63 (d, 2H, *J* = 9 Hz), 9.46 (s, 1H, D_2_O-exchangeable NH), 9.88 (s, 1H, D_2_O-exchangeable NH); ^13^C NMR (DMSO-d_6_): *δ* 28.15, 118.68, 119.93, 123.88, 126.36, 127.85, 128.42, 128.71, 129.20, 129.45, 134.89, 134.97, 138.46, 140.91, 141.32, 145.33, 157.02, 162.59, 189.30; MS* m/z* (%): 492 (M^+^+1, 13.2), 491 (M^+^, 15.1), 320 (28.3), 292 (11.3), 187 (20.8), 105 (100.0), 77 (94.3). Anal. Calcd for C_25_H_21_N_3_O_4_S_2_ (491.59): C, 61.08; H, 4.31; N, 8.55; S, 13.04. Found: C, 61.00; H, 4.24; N, 8.5; S, 13.11%.

### 2.21. 3-Phenyl-5-phenylamino-4-(4-sulfamoylphenyl-carbamoyl)thiophene-2-carboxylic Acid Ethyl Ester (**16**)

Yield (55%), m.p. 280°C (dioxane); IR (KBr) *ν*_max⁡_: 3367, 3310 and 3235 (NH, NH_2_), 3060 (aromatic CH), 1713 (C=O), 1635 (C=O) cm^−1^; ^1^H NMR (DMSO-d_6_): *δ* 1.01 (s, 3H, *J* = 7.2 Hz, CH_3_), 4.04 (q, 2H, *J* = 7.2 Hz, CH_2_), 7.08–7.13 (m, 5H, Ar-H), 7.21 (d, 2H, *J* = 9 Hz), 7.39–7.44 (m, 7H, Ar-H and NH_2_), 7.63 (d, 2H, *J* = 9 Hz), 9.54 (s, 1H, D_2_O-exchangeable NH), 9.73 (s, 1H, D_2_O-exchangeable NH); MS* m/z* (%): 521 (M^+^, 26.3), 322 (15.8), 218 (100.0), 199 (15.8), 77 (86.0). Anal. Calcd for C_26_H_23_N_3_O_5_S_2_ (521.62): C, 59.87; H, 4.44; N, 8.06; S, 12.29. Found: C, 59.77; H, 4.41; N, 8.02; S, 12.21%.

### 2.22. N-[4-(Aminosulfonyl)phenyl]-2-anilino-4-phenyl-5-[(2-oxo-2H-chromen-3-yl)carbonyl]-thiophene-3-carboxamide (**17**)

Yield (55%), m.p. 200°C (EtOH); IR (KBr) *ν*_max⁡_: 3375, 3261 (NH, NH_2_), 1713 (C=O), cm^−1^; ^1^H NMR (DMSO-d_6_): *δ* 6.71–7.27 (m, 7H, Ar-H and NH_2_), 7.29 (d, 2H, *J* = 9 Hz), 7.42–7.59 (m, 10H, Ar-H), 7.63 (d, 2H, *J* = 9 Hz), 9.88 (s, 1H, D_2_O-exchangeable NH), 10.02 (s, 1H, D_2_O-exchangeable NH); MS* m/z* (%): 621 (M^+^, 6.8), 423 (100.0), 250 (15.1), 221 (8.2), 199 (8.2), 145 (24.7), 105 (65.8), 77 (87.7). Anal. Calcd for C_33_H_23_N_3_O_6_S_2_ (621.70): C, 63.76; H, 3.73; N, 6.76; S, 10.32. Found: C, 63.70; H, 3.61; N, 6.67; S, 10.25%.

### 2.23. 2-Benzoyl-3-methylsulfanyl-3-phenylamino-N-(4-sulfamoylphenyl)acrylamide (**18**)

Yield (30%), m.p. 260°C (dioxane); IR (KBr) *ν*_max⁡_: 3343, 3242 (NH, NH_2_), 1696 (C=O) cm^−1^; ^1^H NMR (DMSO-d_6_): *δ* 3.56 (s, 3H, SCH_3_), 7.24 (d, 2H, *J* = 9 Hz), 7.34–7.71 (m, 12H, Ar-H and NH_2_), 7.76 (d, 2H, *J* = 9 Hz), 10.50 (s, 2H, D_2_O-exchangeable 2NH); MS* m/z* (%): 467 (M^+^, 22.9), 296 (45.7), 197 (25.7), 139 (22.9), 105 (14.3), 92 (54.3), 63 (100). Anal. Calcd for C_23_H_21_N_3_O_4_S_2_ (467.56): C, 59.08; H, 4.53; N, 8.99; S, 13.72. Found: C, 59.12; H, 4.50; N, 8.96; S, 13.66%.

### 2.24. 2-(5-Acetyl-3-phenyl-3H-[1,3,4]thiadiazol-2-ylidene)-3-oxo-3-phenyl-N-(4-sulfamoylphenyl)propionamide (**20**)

Yield (35%), m.p. 220°C (dioxane); IR (KBr) *ν*_max⁡_: 3340, 3244 (NH, NH_2_), 3061 (aromatic CH), 1614 (C=O) cm^−1^; ^1^H NMR (DMSO-d_6_): *δ* 2.49 (s, 3H, CH_3_), 7.15 (s, 2H, D_2_O-exchangeable NH_2_), 7.30 (d, 2H, *J* = 9 Hz), 7.62 (d, 2H, *J* = 9 Hz), 7.65–8.39 (m, 10H, Ar-H), 11.95 (s, 1H, D_2_O-exchangeable NH); MS* m/z* (%): 522 (M^+^+2, 14.6), 520 (M^+^, 41.6), 320 (27.0), 306 (13.9), 247 (10.2), 214 (7.3), 189 (8.8), 114 (7.3), 105 (20.4), 77 (100.0). Anal. Calcd for C_25_H_20_N_4_O_5_S_2_ (520.59): C, 57.68; H, 3.87; N, 10.76; S, 12.32. Found: C, 57.63; H, 3.82; N, 10.71; S, 12.29%.

### 2.25. Agar Diffusion Well Method to Determine the Antimicrobial Activity

The microorganism inoculums were uniformly spread using sterile cotton swab on a sterile Petri dish malt extract agar (for fungi) and nutrient agar (for bacteria). One hundred *μ*L of each sample was added to each well (10 mm diameter holes cut in the agar gel, 20 mm apart from one another). The systems were incubated for 24–48 hours at 37°C (for bacteria) and at 28°C (for fungi). After incubation, the microorganism's growth was observed. Inhibition of the bacterial and fungal growth was measured in mm. Tests were performed in triplicate [31].

## 3. Results and Discussion

**Scheme 1:**
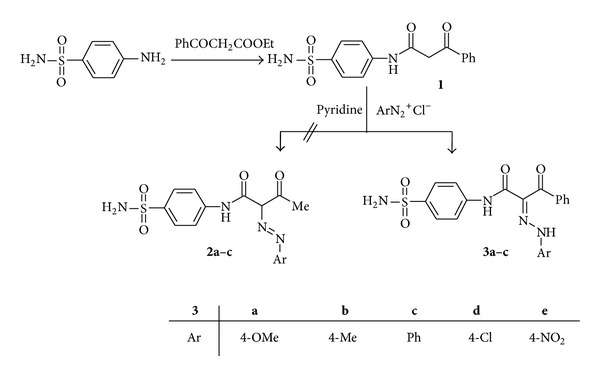
Synthesis of hydrazones derivatives.

Heterocyclic azo compounds are well known for their use as antineoplastics [32], antidiabetics [33], antiseptics [34], and antibacterial activity [35] and are known to be involved in a number of biological reactions such as inhibition of DNA, RNA, protein synthesis, carcinogenesis, and nitrogen fixation [34–36]. Thus, propionamide** 1** was coupled smoothly with diazonium salts, derived from the appropriate aromatic amines [4-methoxyaniline, 4-methylaniline, aniline, 4-chloroaniline, and 4-nitroaniline) in pyridine, to afford the respective hydrazones** 3a**–**e** (Scheme 1). The structures of the products were established on the basis of their elemental analyses and spectral data (IR, ^1^H NMR, and MS) [see Experimental Part]. In the ^1^H NMR spectra of compounds** 3a**–**e**, absence of signal assignable to azomethine group (CH–N=N–) [37] at *δ* 3.00–4.00 ppm ruled out azo form and supported the hydrazone structure of the products.

**Scheme 2:**
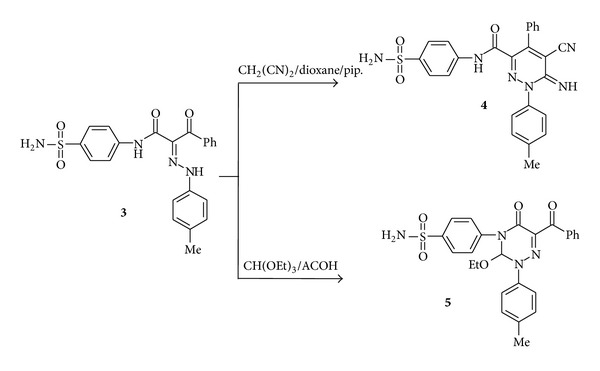
Synthesis of 1,6-dihydropyridazine and 2,3-dihydro-1,2,4-triazine derivatives.

**Scheme 3:**
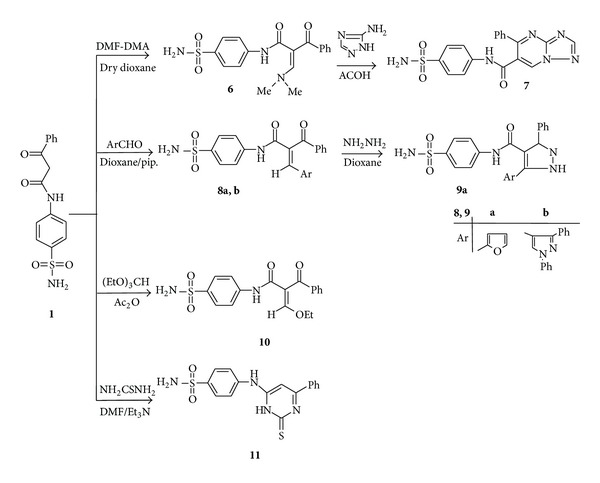


Further elucidation of the structure of** 3b** came from the reaction with malononitrile and triethyl orthformate to furnish the final isolable products corresponding to the 5-cyano-6-imino-4-phenyl-1-*p*-tolyl-1,6-dihydropyridazine-3-carboxylic acid (4-sulfamoylphenyl)amide (**4**) and 4-(6-benzoyl-3-ethoxy-5-oxo-2-*p*-tolyl-2,3-dihydro-1,2,4-triazin-4-(5*H*)-yl)benzenesulfonamide (**5**), respectively. The structures of compounds** 4** and** 5** were confirmed based on elemental analysis and spectral data studied (Scheme 2 and Experimental Part). Treatment of compound** 1** with* N,N*-dimethylformamide-dimethylacetal (DMF-DMA) in refluxing dry dioxane afforded 2-benzoyl-3-dimethylamino-*N*-(4-sulfamoylphenyl)acrylamide (**6**). The ^1^H NMR spectrum of compound** 6** showed signals at *δ* 3.42, 8.10, and 9.98 due to* N,N*-dimethylamino, C=CH–N, and amide-NH protons, respectively. When compound** 6 **was treated with 3-amino-1,2,4-triazole in acetic acid under reflux this led to formation of 1,2,4-triazolo [1,5-a] pyrimidine derivative** 7** (Scheme 3). The IR spectrum of the isolated product showed absorption bands at 3261 and 3110 cm^−1^ characteristic for NH and NH_2_ function. Its ^1^H NMR spectrum showed signals at *δ* 7.68, 8.09, and 8.48 corresponding to triazole-H, pyrimidine-H, and D_2_O-exchangeable signal corresponding to NH protons, respectively. The pathway of formation of product** 7** involves Michael addition of the exocyclic amino group of the heteroamines to the enaminone double bond of** 6**, followed by* in situ* tandem elimination of dimethylamine and dehydrative cyclization.

Also,** 1** reacts with aromatic aldehydes to afford the corresponding 2-benzoyl-3-aryl-2-yl-*N*-(4-sulfamoylphenyl)acrylamide derivatives** 8a**,**b** (Scheme 3). The IR spectrum of compound** 8a**, taken as a typical example, revealed absorption bands at 3349, 3255, and 1687 cm^−1^ corresponding to NH, NH_2,_ and CO functions, respectively. Its ^1^H NMR spectrum showed signals at *δ* 8.48 and 9.14 corresponding to CH and NH protons in addition to aromatic protons at *δ* 7.46–7.94. Its mass spectrum showed a molecular ion peak at* m/z* 396. When the acrylamide derivative** 8a** was treated with hydrazine hydrate it afforded the corresponding pyrazole derivative** 9a** (Scheme 3). Spectroscopic data as well as elemental analyses of the obtained products were in complete agreement with the assigned structures** 9a**.

On the other hand, the reactivity of propionamide** 1** towards triethylorthoformate and thiourea was investigated. Thus, condensation of** 1** with triethylorthoformate in refluxing acetic anhydride afforded the ethoxy methylene derivative** 10**. Establishing of structure** 10** was based on the elemental analysis and spectral data. Treatment of** 1** with thiourea afforded the pyrimidine derivative** 11**. Establishing of compound** 11** is based on its elemental analysis and spectral data (IR and ^1^H NMR) (Scheme 3).

**Scheme 4:**
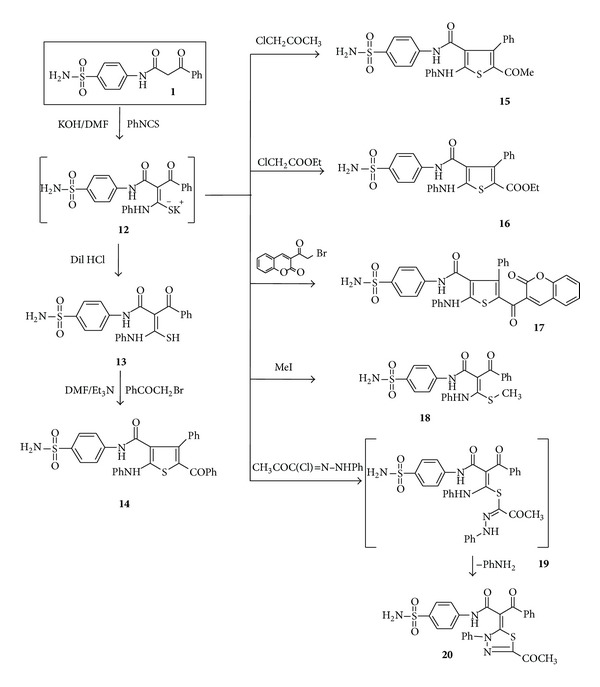


Next, the nucleophilic addition of** 1** to phenyl isothiocyanate in dimethylformamide, in the presence of potassium hydroxide, afforded the corresponding potassium salt** 12**. When the intermediate potassium salt was treated with dilute HCl, it gave the corresponding 2-benzoyl-3-mercapto-3-phenylamino-*N*-(4-sulfamoylphenyl)acrylamide (**13**) (Scheme 4).

The IR spectrum of compound** 13** revealed the absorption bands at 3350–3256 and 1689–1661 cm^−1^ corresponding to NH, NH_2,_ and CO groups, respectively. Its ^1^H NMR spectrum showed two D_2_O-exchangeable signals at 10.39, 11.13, and 12.76 corresponding to 2NH and SH proton, respectively. Moreover, the mass spectrum of the product** 13** exhibited a molecular ion peak at* m/z* 453. Treatment of compound** 13** with 2-bromo-1-phenylethanone in dimethylformamide, in the presence of a catalytic amount of triethylamine, afforded the carboxamide** 14** (Scheme 4). The structure of compound** 14** was elucidated from its spectroscopic and elemental analytical data. Thus, it showed absorption bands at 3425–3111 and 1698–1658 cm^−1^ due to NH, NH_2_, and CO functions, whereas its ^1^H NMR spectra revealed two D_2_O-exchangeable signals at 9.55 and 9.98 corresponding to 2NH protons. Heterocyclisation of the intermediate with chloroacetone or ethyl 2-chloroacetate and 3-(2-bromoacetyl)-2*H*-chromen-2-one furnished in each case one isolable product (as tested by TLC). The reaction products were identified as* N*-[4-(aminosulfonyl)phenyl]-2-anilino-4-phenylthiophene-5-acetyl-3-carboxami-de (**15**), 3-phenyl-5-phenylamino-4-(4-sulfamoylphenyl-carbamoyl)thiophene-2-carboxylic acid ethyl ester (**16**), and* N*-[4-(aminosulfonyl)phenyl]-2-anilino-4-phenyl-5-[(2-oxo-2*H*-chromen-3-yl)carbonyl]thiophene-3-carboxamide (**17**). The reaction proceeds* via* nucleophilic displacement of bromide to give* S*-alkylated intermediate followed by loss of water of the latter intermediate to give thiophene derivatives** 15** or** 16** or** 17** as the final products. The structures of the products** 15**–**17** were determined from spectroscopic and elemental analytical data. Thus, compound** 15**, taken as a typical example, showed absorption bands at 3371, 3289, 3220, and 1636 cm^−1^ corresponding to NH, NH_2,_ and C=O groups, respectively. Its ^1^H NMR spectrum revealed the absence of CH_2_ protons of chloroacetone and showed signals at *δ* 9.46 and 9.88 due to 2NH protons, in addition to an aromatic multiplet in the region *δ* 7.13–7.63. The ^13^C NMR of compound** 15** revealed signals at 28.15, 162.59, and 189.30 for the carbons of the CH_3_ of (COCH_3_), CO of (CONH), and CO of (COCH_3_) groups.

Furthermore, the nonisolated potassium salt was methylated by treatment with methyl iodide to afford the novel ketene* N*,* S*-acetal** 18**. The structure of the synthesized product was established on the basis of their elemental analysis and spectral data [see the Experimental Part].

Heterocyclisation of the intermediate** 12** with 2-oxo-*N*-phenylpropane hydrazonoyl chloride [38] furnished one isolable product (as tested by TLC). The reaction product was identified as 2-(5-acetyl-3-phenyl-3*H*-[1,3,4]-thiadiazol-2-ylidene)-3-oxo-3-phenyl-*N*-(4-sulfamoylphenyl)propionamide (**20**) (Scheme 4). The structure of the product** 20** was determined from spectroscopic and elemental analytical data. Thus, the IR spectrum of compound** 20** revealed absorption bands at 3340, 3244, and 1614 cm^−1^ corresponding to NH, NH_2,_ and CO groups, respectively. Its ^1^H NMR spectrum revealed signals at *δ* 2.49 and 11.95 due to CH_3_ and NH protons, in addition to an aromatic multiplet in the region *δ* 7.30–8.39. The aforementioned results indicate that the reaction proceeds* via S*-alkylation [39], to give* S*-alkylated intermediate** 19** which cyclized* in situ* under the employed reaction conditions and elimination of aniline molecule gave the desired product** 20** (Scheme 4).

*Screening for Antimicrobial Activity.* The newly synthesized compounds** 3a**,** 3c**,** 6**,** 7**,** 8b**,** 10**,** 15**,** 16**, and** 17** were evaluated for their* in vitro* antibacterial activity against* Staphylococcus aureus* (RCMB-000106) (*SA*) and* Bacillus subtilis* (RCMB-000108) (*BS*) as examples of Gram-positive bacteria and* Pseudomonas aeruginosa* (RCMB-000102) (*PA*) and* Escherichia coli* (RCMB-000103) (*EC*) as examples of Gram-negative bacteria. They were also evaluated for their* in vitro* antifungal activity against* Aspergillus fumigatus* (RCMB-002003) (*AF*),* Saccharomyces cerevisiae* (RCMB-006002) (*SC*), and* Candida albicans* (RCMB-005002) (*CA*) fungal strains. Inhibition zone diameter (IZD) in mm was used as criterion for the antimicrobial activity using the agar diffusion well method. The fungicide clotrimazole and the bactericide* streptomycin* were used as references to evaluate the potency of the tested compounds under the same conditions. The results are depicted in Table 1. From the data given by Table 1 we concluded that most of the tested compounds displayed variable degrees of antibacterial activity against Gram-positive bacteria, Gram-negative bacteria strains, and also against fungal strains in comparison to the standard in each case which revealed that these compounds are biologically active. Compound** 7** exhibited high degree of antibacterial activity against Gram-positive bacteria (*SA*). Compounds** 3c** and** 7** have moderate degree of antibacterial activity against Gram-negative bacteria (*EC*) and (*PA*). All the compounds exhibited moderate antifungal activity against (*AF*) and high activity against (*SC*,* CA*). The structure antimicrobial activity relationship of the synthesized compounds revealed that the maximum activity was attained with compound** 15**, having acetyl thiophene moiety.

## 4. Conclusions

Several new hydrazones, pyridazines, triazines, acrylamides, pyrazoles, triazolo[1,5-a]pyrimidines, thiadiazoles, dihydropyrimidines, and thiophenes containing sulfamoyl moiety were prepared using simple methods* via* a versatile; readily accessible 3-oxo-3-phenyl-*N*-(4-sulfamoylphenyl)propionamide (**1**) is demonstrated. The structures of the newly synthesized compounds were proven by spectral methods and they were tested for their antimicrobial activities. Most of these compounds showed promising activities against both Gram-positive, Gram-negative bacteria and fungi.

## Figures and Tables

**Table 1:** Antimicrobial and Antifungal Activities of the Synthesized Compounds.

Sample no.	Inhibition zone diameter (mm)						
	Gram (−)		Gram (+)		Fungi		
	(PA)	(EC)	(SA)	(BS)	(AF)	(SC)	(CA)
**3a**	10.7 ± 0.3	10.1 ± 0.4	12.2 ± 0.3	11.1 ± 0.4	11.9 ± 0.4	12.5 ± 0.4	13.4 ± 0.4
**3c**	12.9 ± 0.4	11.7 ± 0.4	12.1 ± 0.4	12.3 ± 0.4	18.7 ± 0.4	20.8 ± 0.6	14.7 ± 0.5
**6**	10.6 ± 0.3	10.8 ± 0.4	12.2 ± 0.4	11.3 ± 0.4	17.1 ± 0.5	17.9 ± 0.6	14.8 ± 0.4
**7**	13.4 ± 0.4	13.1 ± 0.4	19.7 ± 0.4	20.1 ± 0.5	17.6 ± 0.4	18.8 ± 0.5	13.9 ± 0.5
**8b**	11.1 ± 0.3	12.7 ± 0.4	11.8 ± 0.3	11.5 ± 0.4	15.3 ± 0.5	19.1 ± 0.4	15.2 ± 0.5
**10**	9.7 ± 0.2	8.9 ± 0.4	11.2 ± 0.4	10.8 ± 0.4	12.1 ± 0.5	11.9 ± 0.4	11.7 ± 0.5
**15**	24.3 ± 0.1	25.6 ± 0.1	25.1 ± 0.5	30.1 ± 0.6	26.1 ± 0.5	23.1 ± 0.4	18.3 ± 0.6
**16**	11.1 ± 0.4	10.3 ± 0.3	9.8 ± 0.3	10.9 ± 0.4	11.4 ± 0.3	12.2 ± 0.4	11.3 ± 0.5
**17**	9.2 ± 0.3	7.8 ± 0.4	8.9 ± 0.3	9.7 ± 0.3	10.5 ± 0.4	10.3 ± 0.4	9.5 ± 0.4
*Clotrimazole *	—	—	—	—	26.1 ± 0.5	23.1 ± 0.4	18.3 ± 0.6
*Streptomycin *	—	—	25.1 ± 0.5	30.1 ± 0.6	—	—	—
*Streptomycin *	24.3 ± 0.4	25.6 ± 0.5	—	—	—	—	—

Data are expressed in the form of mean ± SD. Mean zone of inhibition in mm ± Standard deviation beyond well diameter (6 mm) produced on a range of environmental and clinically pathogenic microorganism using (5 mg/mL) concentration of tested sample (100 *μ*L was tested).
